# Obesity and high waist circumference are associated with low circulating pentraxin-3 in acute coronary syndrome

**DOI:** 10.1186/1475-2840-12-167

**Published:** 2013-11-11

**Authors:** Rocco Barazzoni, Aneta Aleksova, Cosimo Carriere, Maria Rosa Cattin, Michela Zanetti, Pierandrea Vinci, Davide Stolfo, Gianfranco Guarnieri, Gianfranco Sinagra

**Affiliations:** 1 Clinica Medica-Department of Medical, Surgical and Health Sciences University of Trieste, Trieste, Italy; 2 Cardiovascular Department, “Ospedali Riuniti” and University of Trieste, Trieste, Italy

**Keywords:** Obesity, Pentraxin-3, Acute coronary syndrome

## Abstract

**Background:**

Long pentraxin 3 (PTX3) is a component of the pentraxin superfamily and a potential marker of vascular damage and inflammation, associated with negative outcome in patients with acute coronary syndromes (ACS). Obesity is a risk factor for cardiovascular disease and PTX3 production is reported in abdominal adipose tissue. Low PTX3 is however reported in the obese population, and obesity per se may be associated with less negative ACS outcome.

**Methods:**

We investigated the potential impact of obesity and high waist circumference (reflecting abdominal fat accumulation) on plasma PTX3 concentration in ACS patients (n = 72, 20 obese) compared to age-, sex- and BMI-matched non-ACS individuals.

**Results:**

Both obese and non-obese ACS patients had higher PTX3 than matched non-ACS counterparts, but PTX3 was lower in obese than non-obese individuals in both groups (all P < 0.05). PTX3 was also lower in ACS subjects with high than in those with normal waist circumference (WC). Plasma PTX3 was accordingly associated negatively with BMI and WC, independently of age and plasma creatinine. No associations were observed between PTX3 and plasma insulin, glucose or the short pentraxin and validated inflammation marker C-reactive protein, that was higher in ACS than in non-ACS individuals independently of BMI or WC.

**Conclusions:**

Obesity is associated with low circulating PTX3 in ACS. This association is also observed in the presence of abdominal fat accumulation as reflected by elevated waist circumference. Low PTX3 is a novel potential modulator of tissue damage and outcome in obese ACS patients.

## Introduction

The pentraxin superfamily includes short and long components [1,2]. C-reactive protein is a liver-synthesized short pentraxin and a strongly validated marker of systemic inflammation [1-3]. Long pentraxins are however synthesized by various cell types and may differentially modulate the inflammatory response under different clinical conditions [1-3]. In particular, long pentraxin 3 (PTX3) may be secreted by adipocytes under pro-inflammatory stimuli and it has been proposed as a clinical marker of vascular damage [1,2]. Plasma PTX3 was accordingly reported to be elevated in patients with arterial stiffness [4] and subclinical [5] or unstable atherosclerotic lesions [6], and high circulating PTX3 is observed in acute coronary syndromes (ACS) [7,8]. In ACS patients, higher PTX3 was also remarkably associated with negative outcome in terms of subsequent events and overall survival [9,10]. Despite its clinical relevance, factors modulating circulating PTX3 in ACS remain however incompletely defined.

Obesity is an independent risk factor for coronary artery disease and ACS, but obesity per se has been paradoxically associated with improved prognosis in ACS patients [11,12]. In the general population [4,13] and in disease states including chronic kidney failure [14,15] and insulin resistance or metabolic syndrome [16-19], low plasma PTX3 was found in most reports in obese individuals and in subjects with high waist circumference, despite high PTX3 expression in abdominal fat [20,21]. The potential interactions between obesity, abdominal fat accumulation and ACS in modulating plasma PTX3 remain to be defined. In the current study we therefore investigated the impact of obesity and waist circumference on plasma PTX3 in non-obese and obese ACS patients and in sex-, age and BMI-matched non-ACS control subjects. We hypothesized that obesity has a negative impact on circulating PTX3 in ACS, and that similar interactions are also observed between PTX3 and high waist circumference, a surrogate marker of abdominal fat accumulation. Finally, we tested the hypothesis that changes in PTX3 are unrelated to those of the short pentraxin and inflammation marker CRP in ACS patients.

## Methods and procedures

### Subjects and experimental protocol

The study conforms to the principles outlined in the Declaration of Helsinki and was approved by the institutional Ethics Committee. All patients were given detailed information on the study aims and risks and they gave written consent before enrolled. In all participants, clinical history and complete physical examination including measurements of blood pressure, body mass index (BMI) and waist circumference were collected. Obesity was defined as BMI > 30 kg/m2, while high waist circumference was defined based on Adult Treatment Panel III diagnostic criteria for metabolic syndrome (>102 or 88 cm for male and female subjects, respectively). Diagnosis of hypertension was based on blood pressure measurement (>135/85 mmHg) or antihypertensive medications; diagnosis of dyslipidemia was based on plasma triglycerides (>150 mg/dl) and HDL cholesterol (<50 or 40 mg/dl for females and males respectively) or triglyceride-lowering medications; diagnosis of type 2 diabetes was based on HbA1c >6.5% or antidiabetic medications. Exclusion criteria were clinical or laboratory evidence of liver failure or disease, renal failure (plasma creatinine above 1,5 mg/dl), cancer, chronic autoimmune and thyroid disease. Females taking hormonal estrogen therapy were also excluded from the study. No subject in either group had history or clinical or laboratory signs of systemic inflammatory disease.

#### ACS

72 consecutive patients with acute coronary syndrome (50 non-obese, 22 obese) were recruited in Coronary Care Unit from the Cardiovascular Department of the Azienda Ospedaliero-Universitaria “Ospedali Riuniti” in Trieste. ACS was diagnosed based on WHO criteria in the presence of two of the following criteria: ischemic chest pain, serial ECG modifications, troponin I elevation with subsequent reduction. For all patients, one overnight fasted blood sample was collected within 24 hours of admission. No differences in timing of sample collection occurred between non-obese and obese patients. After separation, plasma was stored at −80 C until biochemical and hormonal measurements were performed.

#### Non-ACS

52 control subjects with no clinical history of coronary artery disease based on detailed history and clinical examination were also studied (33 non-obese, 19 obese). These subjects were matched to the ACS groups for age, sex, BMI, waist circumference. Overnight-fasted blood samples were collected also for control subjects. Part of the study results in a smaller study population, pertaining to the associations between obesity, ACS, insulin resistance and adipose tissue hormones have been reported elsewhere [22].

### Plasma analyses

Plasma glucose, HDL cholesterol and plasma triglycerides were measured using standard methods. Plasma insulin was measured by ELISA (Insulin Human Ultrasensitive ELISA; DRG Instruments, Marburg, Germany). Insulin sensitivity was assessed by the validated HOMA index using the following formula: HOMA = (FPG*FPI)/22.5, where FPG and FPI are fasting plasma glucose (mmol) and fasting plasma insulin (μU/ml) respectively [20]. Plasma pentraxin-3 (PTX3) (Human Pentraxin3/TSG-14ELISA System Perseus Proteomics Inc., Tokyo, Japan) and C-reactive protein (CRP) (High sensitivity c-reactive protein, Diagnostics Biochem Canada Inc London, Ontario, Canada) were measured using commercially available ELISA kit.

### Statistical analysis

The StatView software (SAS Institute, Cary, NC, USA) was used for statistical analyses. Normality Tests were run to assess data distribution. Comparisons between ACS patients and non-ACS control subjects were made by unpaired Student’s t-test or Wilcoxon test for non-parametric analyses in variables with non-normal distribution (PTX3, HOMA-IR and CRP). To assess differences between obese and non-obese ACS and non-ACS patients, ANOVA or Kruskal-Wallis test for non-parametric variables were used. Linear regression analysis was used to determine associations between PTX3 and different study variables that are potentially involved in its regulation. Multiple regression analysis was then used to investigate potential independent associations between groups of statistically related variables. Due to non-normal distribution, log-transformed values for PTX3, HOMA-IR and CRP were used for regression analyses, and log-transformed PTX3 was used as dependent variable in multiple regression analyses. All data are reported as Mean ± Standard Deviation and range, unless stated otherwise. P values of less than 0.05 were considered statistically significant.

## Results

### Clinical characteristics, metabolic and hormonal profile

ACS and non-ACS patients were comparable for sex, age, BMI, waist circumference, prevalence of hypertension and dyslipidemia, type 2 diabetes, blood pressure, lipid profile, plasma glucose and HOMA insulin resistance index. Plasma C-reactive protein and PTX3 were higher in the whole ACS group than in non-ACS patients (Table 1).

**Table 1:** Clinical and biochemical profile

	**ACS**	**Control**
**Gender (M/F)**	60/12	44/8
**Age (years)**	63 ± 10 (42–85)	62 ± 5 (54–73)
**BMI (kg/m2)**	27.5 ± 3.8 (20.6-40.8)	27.9 ± 3.6 (21.6-40.5)
**WC (cm)**	101 ± 10 (70–137)	100 ± 9 (80–118)
**Prevalence (%)**		
**Hypertension**	68	62
**Dyslipidemia**	70	62
**Type 2 Diabetes**	16	12
**Medications (%)**		
**ACE-Inhib or ARB**	48	42
**Ca-Channel Blocker**	18	20
**Beta-Blockers**	44	38
**Diuretics**	16	20
**Statins**	34	30
**ASA**	38	24
**SBP (mmHg)**	131 ± 18 (95–175)	136 ± 16 (115–165)
**DBP (mmHg)**	72 ± 10 (55–100)	76 ± 6 (70–95)
**Creat (mg/dl)**	1.01 ± 0.28 (0.55-1.5)	0.92 ± 0.20 (0.60-1.4)
**Tg (mg/dl)**	126 ± 65 (44–585)	146 ± 35 (110–289)
**T-Chol (mg/dl)**	191 ± 42 (81–316)	215 ± 41 (127–261)
**HDL-Chol (mg/dl)**	45 ± 8 (24–85)	48 ± 8 (32–65)
**Glucose (mg/dl)**	121 ± 26 (60–221)	114 ± 10 (86–131)
**Insulin (**μ**U/ml)**	4.6 ± 2.4 (1.5-12)	5.4 ± 3.3 (1.2-15.4)
**HOMA-IR**	1.45 ± 0.95 (0.3-4.8)	1.50 ± 0.96 (0.5-3.8)
**CK (mU/ml)**	124 (8.4-888)	-
**Troponin (ng/ml)**	14.6 (0.5-326)	-
**CRP (mg/dl)**	2 (0.2-15)	0.2 (0.02-3.1)*
**PTX3 (ng/ml)**	5.7 (1.8-29.3)	2.7 (1.2-5.6)*

Gender, age, BMI, waist circumference (WC), disease prevalence, chronic medications (treatment before event for ACS); ACE-Inhib: Angiotensin Converting Enzyme-Inhibitors; ARB: Angiotensin Receptor Blockers; ASA: Acetylsalicylic Acid; systolic (SBP) and diastolic (DBP) blood pressure, plasma creatinine (creat), triglycerides (Tg), HDL cholesterol (HDL-Chol), glucose, insulin, Homeostasis Model Assessment (HOMA), plasma C-reactive protein (CRP) and pentraxin-3 (PTX3) in ACS and Control groups. Results are reported as Mean ± SD (Range) except for non-normally distributed HOMA-IR, CRP, PTX3, that are presented as Median (Range). *:P < 0.05 between ACS and Control groups by Wilcoxon test for non-normally distributed data (HOMA-IR, CRP, PTX3).

### Obesity, waist circumference and PTX3

**Figure 1:**
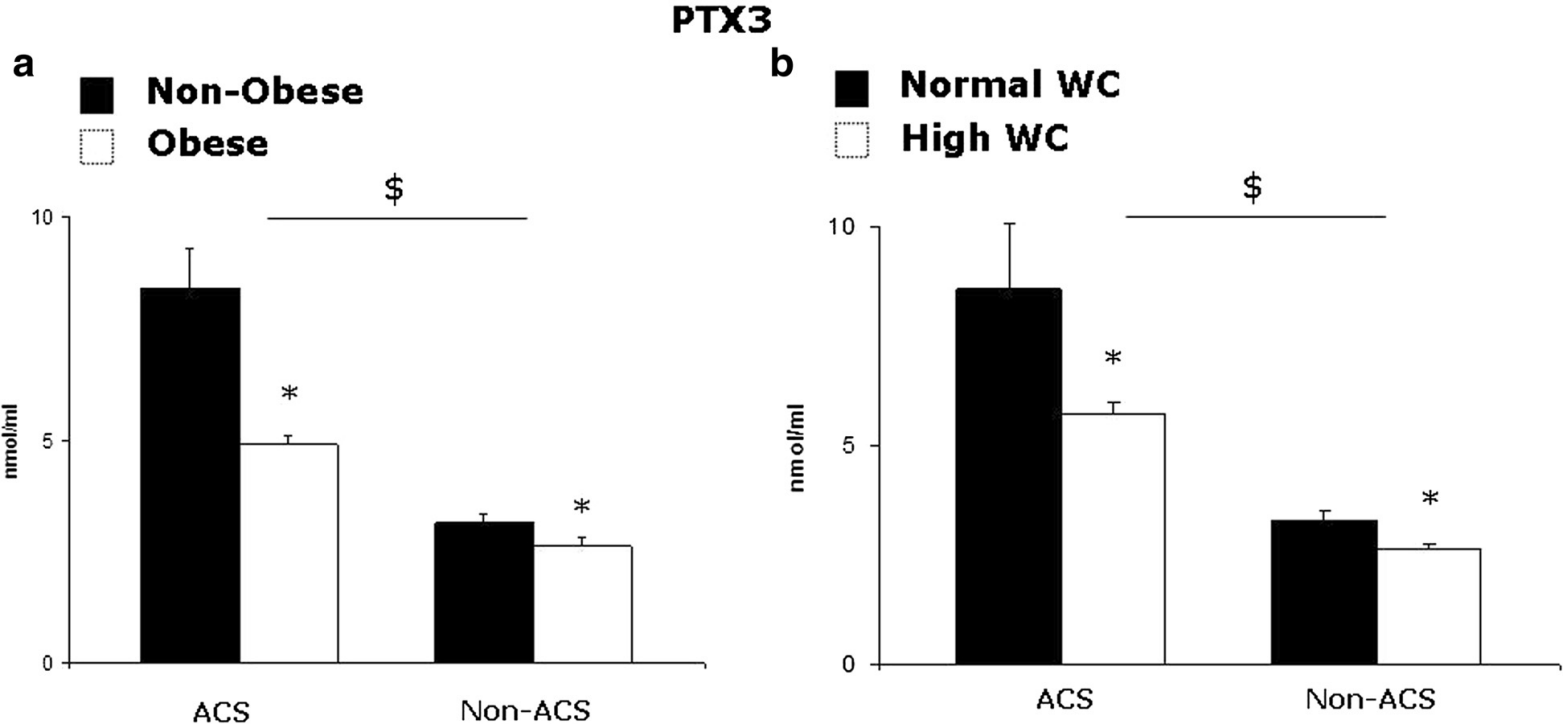
**a) Plasma PTX3 in non-obese and obese (BMI > 30 kg/m**^**2**^**) acute coronary syndrome patients (ACS; non-obese n = 49, obese n = 23) and control subjects (non-ACS; non-obese n = 33, obese n = 19); b) Plasma PTX3 in patients with normal or high waist circumference (WC) (> 102 or 88 cm for male and female subjects, respectively) in the acute coronary syndrome (ACS; normal WC n = 34, high WC n = 38) and control groups (non-ACS; normal WC n = 23, high-WC n = 29).** *: P < 0.05 Obese vs corresponding Non-Obese group; $: P < 0.05 Obese and Non-Obese ACS vs corresponding Non-ACS group, by Kruskal-Wallis test.

Both non-obese and obese subjects with ACS had higher PTX3 compared to matched control subjects (P < 0.05). PTX3 was however lower in obese than in non-obese individuals in both ACS (Non-Obese: 8.4 ± 0.9, Obese: 4.8 ± 0.1 nmol/ml, P = 0.02) and non-ACS group (Non-Obese: 3.2 ± 0.1, Obese: 2.5 ± 0.1, P = 0.04) (Figure 1a). Similarly, when subjects were divided into two groups with high or normal waist circumference according to ATP III classification (Males: WC > 102 cm, Females: WC > 88 cm), PTX3 was lower in high- than in normal-waist circumference individuals in both ACS (Normal WC: 8.7 ± 1.1, High WC: 5.6 ± 0.24 nmol/ml, P = 0.046) and non-ACS group (Normal WC: 3.3 ± 0.14, High WC: 2.6 ± 0.1 nmol/ml, P = 0.03) (Figure 1b).

### Linear regression analysis between PTX3 and anthropometric and biochemical variables in all ACS subjects

**Figure 2:**
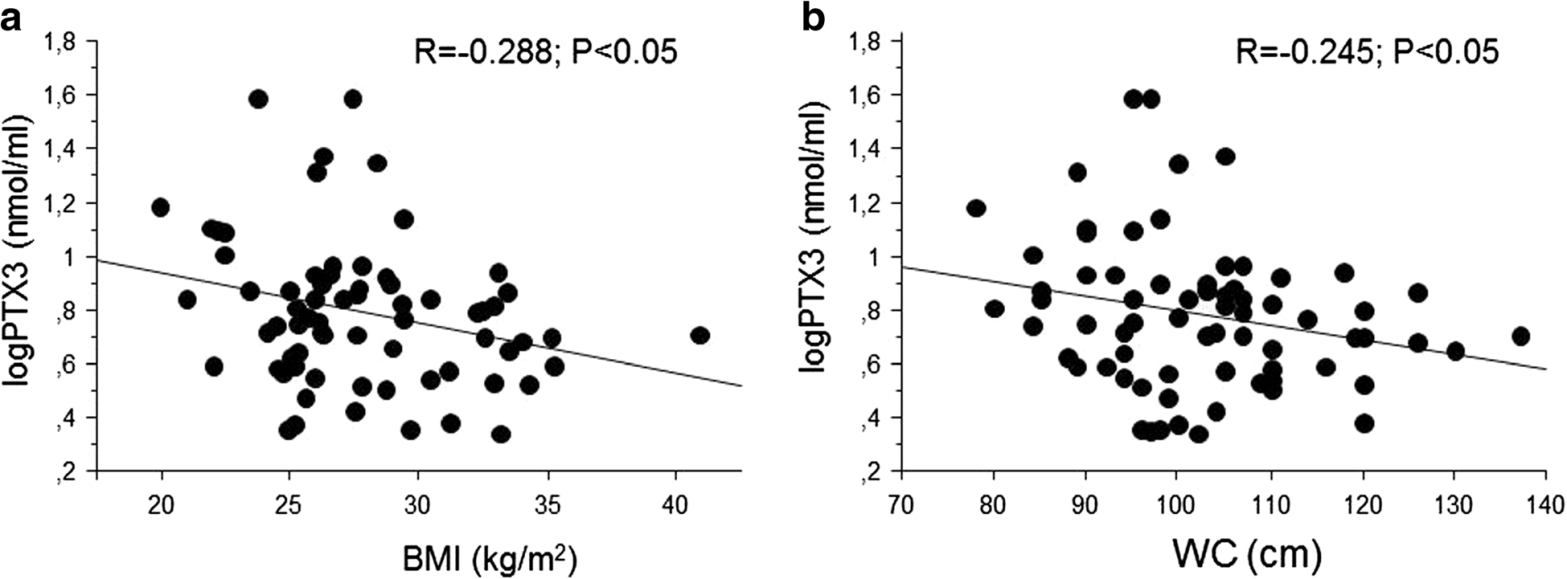
Associations between plasma PTX3 and BMI (a) or WC (b) in ACS patients (n = 72).

In all ACS subjects (n = 72) PTX3 was associated positively with age and plasma creatinine. Consistent with the impact of obesity and waist circumference on PTX3, plasma PTX3 was associated negatively with BMI and WC (Figure 2), and both associations were independent of age and plasma creatinine in multiple regression analysis (Tables 2, 3). No statistically significant associations were instead observed between PTX3 and total and HDL-cholesterol, triglycerides, blood pressure, plasma glucose, insulin and HOMA insulin resistance index (Table 2), despite higher insulin and HOMA index in obese compared to non-obese subjects in both ACS and control groups (P < 0.05). Similar associations of PTX3 were observed in the control group alone (BMI: r = −0.32, P = 0.04, WC: r = −0.22, P = 0.08). When all patients and control subjects were considered together, however, the correlations were no longer significant (BMI: r = −0.15, P = 0.11, WC: r = −0.04, P > 0.2) likely due to different PTX3 plasma concentrations in the two groups at any given BMI level.

**Table 2:** Linear regression analysis between PTX3 as dependent variable and age, plasma creatinine, triglycerides, total and HDL-cholesterol, glucose, insulin, HOMA index and plasma C-reactive protein (CRP) in all ACS patients (n = 72)

	**PTX3**
	**r**
**Age** (years)	0.254^*^
**Creatinine** (mg/dl)	0.338^*^
**Tg** (mg/dl)	0.121
**T-Chol** (mg/dl)	0.075
**HDL-Chol** (mg/dl)	0.022
**Insulin** (μU/ml)	0.081
**Glucose** (mg/dl)	0.038
**HOMA**	0.048
**CK** (mU/ml)	0.090
**Troponin** (ng/ml)	0.034
**CRP** (mg/dl)	0.086

*:P < 0.05.

**Table 3:** Multiple regression analyses between PTX3 (dependent variable) and variables associated with PTX3 in linear regression analysis: age, plasma creatinine, BMI (independent variables), in all ACS patients (n = 72)*: P < 0.05; **: P < 0.01

	**PTX3**	
	**β value**	**STD Error**
**Age**	0.004	0.003
**Creatinine**	0.162^**^	0.008
**BMI**	−0.016^*^	0.057

Similar results were observed when WC was used instead of BMI (not shown).

*:P < 0.05.

### Obesity, waist circumference and CRP

**Figure 3:**
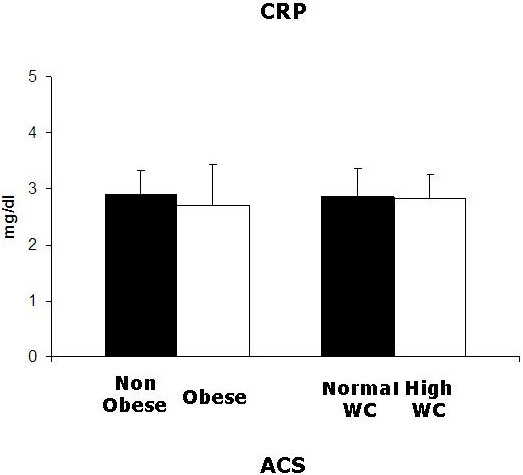
**Plasma CRP in ACS patients.** Left: non-obese (n = 49) and obese (n = 23) patients; right: normal WC (n = 34) or high WC (n = 38) patients. No statistically significant differences were observed by Kruskal-Wallis test.

At variance with plasma PTX3, CRP was not lower in obese than in non-obese ACS patients (P > 0.2). Comparable CRP plasma concentrations were also observed when patients were stratified according to waist circumference (P > 0.2). Plasma CRP was accordingly not associated with BMI or WC in linear regression analysis (P > 0.2, not shown) (Figure 3).

## Discussion

In the current study we provide novel information on the impact of obesity or waist circumference on plasma PTX3 in ACS. Results demonstrate that: 1) ACS leads to plasma PTX3 elevation in both non-obese and obese patients; 2) obesity is however associated with lower PTX3 in people with and without ACS; 3) lower PTX3 is also observed in patients with normal compared to those with high waist circumference, a marker of abdominal fat accumulation.

PTX3 is a component of the pentraxin superfamily reportedly involved in the modulation of vascular inflammation and damage [1,2]. Although the majority of available studies indicate a negative impact of obesity on plasma PTX3 in the general population and various disease states [11-18], obesity is a strong risk factor for cardiovascular events and PTX3 is commonly elevated in ACS [7-10]. The current data confirm that ACS enhances circulating PTX3, but they further demonstrate that a negative impact of obesity on plasma PTX3 extends from non-ACS to ACS individuals. Lack of statistically significant associations indicates that changes in plasma lipid profile, glucose metabolism or systemic inflammation were unlikely to contribute to lower PTX3 in obese ACS patients. Since abdominal adipose tissue is a potential relevant source of PTX3 [20,21], the impact of waist circumference on plasma PTX3 was also directly investigated, and a negative association was also observed in ACS between waist circumference and PTX3. Low PTX3 production in abdominal adipose tissue could therefore be, at least in part, paradoxically responsible for lower PTX3 plasma concentration in ACS patients with high waist circumference. As an alternative explanation, obesity and abdominal fat accumulation could lower PTX3 production in other cell types through yet unidentified signalling and mechanisms, that should be investigated in future studies.

Higher PTX3 is associated with negative outcome in ACS, and the current results therefore suggest that less pronounced PTX3 elevation may contribute to positively modulate outcome and survival in obese ACS patients [7-10]. The association between PTX3 and negative outcome had been originally proposed to involve direct negative effects of PTX3 in cardiac and vascular tissues [7]. Strong emerging evidence however indicates that PTX3 elevation may represent an adaptive, anti-inflammatory response to pre-existing vascular damage [23,24], and this concept is also supported by differential changes of pro-inflammatory short pentraxin CRP and PTX3 in ACS in the current study. More pronounced tissue damage, rather than PTX3 elevation per se, could therefore be directly responsible for negative outcome in ACS patients with highest PTX3. Potential BMI-dependent characteristics of cardiovascular lesions should be directly investigated in obese ACS patients, along with their potential impact on PTX3.

Limitations of the present study should be acknowledged. First, factors regulating PTX3 production and plasma concentration remain largely unknown, and the current cross-sectional study design in vivo could not directly address potential mechanisms underlying altered circulating PTX3, that should be investigated in experimental models. The potential interaction between obesity, PTX3 and ACS outcome and survival will also need to be investigated and confirmed in future studies. Finally, we selected to base the diagnosis of diabetes on HbA1c levels, since plasma glucose could have been acutely affected by metabolic changes induced ACS per se. The current findings however indicate a novel link between obesity and plasma PTX3 in ACS, and understanding the underlying mechanisms will likely lead to novel potential prevention and treatment strategies to improve ACS prognosis in both obese and non-obese patients.

## Conclusion

In conclusion, we demonstrated a negative impact of obesity on circulating PTX3 in ACS patients. A similar negative impact was also observed for elevated waist circumference, a surrogate marker of abdominal fat accumulation. These effects do not extend to the short pentraxin and validated inflammation marker CRP, whose plasma concentrations were not reduced in obese ACS patients. Low PTX3 is a novel potential modulator of tissue damage and outcome in obese ACS patients.

## Competing interests

The authors declare that they have no competing interests.

## Authors’ contributions

RB designed the study, participated in data analyses and drafted the manuscript; AA contributed to design the study, recruited patients, participated in data analyses and in drafting the manuscript; CC recruited patients, participated in data analyses and keeping the patient database; MRC, MZ and PV recruited patients and participated in data analyses and discussion; DS recruited patients, participated in statistical analyses; GG participated in drafting the manuscript; GS designed the study and drafted the manuscript. All authors read and approved the final manuscript.
